# Rehabilitation for Physical Frailty in Lung Transplant Candidates: A Systematic Review

**DOI:** 10.1097/CPT.0000000000000265

**Published:** 2024-12-27

**Authors:** Laura McGarrigle, Gill Norman, Helen Hurst, Loraine Gillespie, Chris Todd

**Affiliations:** 1 Department of Cardiothoracic Transplantation, Manchester University NHS Foundation Trust, Manchester, United Kingdom; 2 Evidence Synthesis Group, Population Health Sciences Institute, Newcastle University, Newcastle Upon Tyne, United Kingdom; 3 NIHR Innovation Observatory, Population Health Sciences Institute, Newcastle University, Newcastle Upon Tyne, United Kingdom; 4 School of Health and Society, University of Salford, Salford, United Kingdom; 5 Northern Care Alliance NHS Foundation Trust, Salford, United Kingdom; 6 Department of Nutrition and Dietetics, The Christie NHS Foundation Trust, Manchester, United Kingdom; 7 School of Health Sciences, Faculty of Biology, Medicine and Health, University of Manchester, Manchester, United Kingdom; 8 NIHR Applied Research Collaboration Greater Manchester, School of Health Sciences, Faculty of Biology, Medicine and Health, University of Manchester, Manchester, United Kingdom; 9 Manchester University NHS Foundation Trust, Manchester, United Kingdom

**Keywords:** exercise, prehabilitation, waiting list

## Abstract

**Purpose:**

Physical frailty is prevalent in lung transplant (LTx) candidates and is linked to adverse outcomes preoperatively and postoperatively. Exercise is beneficial in optimizing exercise capacity and quality of life in candidates, but its impact on physical frailty is unknown.

**Methods:**

We prospectively registered and published a protocol (PROSPERO CRD42022363730) before undertaking a systematic review. We searched 4 databases plus trial registries from 1980 to February 2024 for studies of exercise interventions in adults awaiting LTx. Outcomes were measures or surrogate markers of physical frailty. An NIH assessment tool was used to assess study quality, and certainty of evidence was assessed using GRADE.

**Results:**

Fifteen studies (664 patients) were included. Interventions were in-person pulmonary rehabilitation, home exercise, and telerehabilitation. Studies included aerobic, resistance, balance, and breathing training. Only 2 studies assessed frailty using a phenotypic measure. Studies demonstrated improvement in some surrogate frailty outcomes including the Short Physical Performance Battery, 5 times sit-to-stand test, and handgrip or muscle strength measures. The study quality was fair or poor; evidence was low or very low certainty for all outcomes due to imprecision and high risk of bias. Uncontrolled study designs and heterogeneity of interventions and outcomes limit conclusions on effectiveness.

**Conclusions:**

Exercise training appears beneficial in modifying surrogate markers of physical frailty before LTx, but conclusions are limited by low or very low certainty evidence. High quality randomized trials are needed to determine the impact of exercise interventions on physical frailty and to develop guidelines for LTx prehabilitation.

## Abstract 2

Supplemental Digital Content is Available in the Text.

**Clinical Pearls**

•This is the first review of exercise interventions to modify physical frailty in lung transplant candidates.
•In-person and remote rehabilitation programs incorporating aerobic and strengthening elements appear to show potential in improving measures and surrogate measures of frailty before a lung transplant.
•There is a lack of randomized controlled trials evaluating the effects of exercise interventions on physical frailty before lung transplant; therefore, the certainty of evidence in this review is low to very low. This highlights the need for robust and rigorous methodologies of studies in this field.
•The participant, intervention, and outcome heterogeneity and gaps in reporting prevents clear conclusions being drawn around the optimal intervention to tackle frailty in this population.

## INTRODUCTION

Lung transplantation (LTx) is the process of surgical replacement of lung(s) typically due to end-stage respiratory disease that is unresponsive to maximal medical therapy. Advanced lung disease is associated with dyspnea, limited exercise capacity, disability, and reduced quality of life. A small percentage of individuals with severe, chronic lung disease meet stringent international criteria for LTx.^1^

Frailty is a state characterized by lack of physiological reserve, leaving individuals at increased vulnerability to stressors. It is commonly seen in those with chronic end-stage lung disease including those referred for LTx.^2-4^ The proportion of LTx recipients aged older than 65 years continues to rise^5^ despite increasing age being an independent risk factor of poor outcomes after LTx^6^ and increased incidence of frailty.^7^

Physical frailty has been shown to have a detrimental impact on pre-LTx and post-LTx morbidity and mortality.^4,8^ Physical frailty is associated with an increased postoperative hospital length of stay, disability, reduced health-related quality of life, and increased risk of hospital readmission.^4,9^ Transplant teams are challenged to differentiate chronologic age from functional status and to identify, select, and prepare individuals with the physical and psychological reserve necessary to cope with the demanding transplantation recovery period.^1,6,10^ Teams are therefore increasingly measuring frailty as part of the LTx evaluation of suitability.^6^

Recent systematic reviews have concluded that exercise programs containing aerobic and resistance training before LTx have the capacity to improve the exercise capacity and quality of life with some evidence of increases in muscle strength.^11,12^ Pulmonary rehabilitation (PR), an evidence-based program of exercise interventions and education, is widely recommended for all LTx candidates.^1^ The assumption is that “prehabilitation” addresses modifiable risk factors that allows patients to undergo surgery in a more optimal, less frail physical state, which may potentially reduce postoperative complications, disability, and mortality.^13^ In addition, pulmonary rehabilitation improves fried frailty phenotype (FFP) scores toward a more robust state in the short term in individuals with chronic obstructive pulmonary disease (COPD).^14^ The optimal process of preparation before LTx for a population with a spectrum of lung conditions is still not fully understood,^1,10^ and the evidence for prehabilitation on LTx outcomes is not conclusive. Further work is required to establish the effectiveness of interventions to tackle physical frailty; refine candidate selection processes; improve survival, function, and quality of life; and therefore, maximize the benefit of LTx from such a limited pool of donor organs.^1,3,10,15,16^

Elements of the review were defined using the recognized participant, intervention, comparator, outcome (PICO) framework. The objectives of this study were to systematically evaluate the effectiveness of exercise (intervention) in modifying physical frailty (outcome) in adults awaiting lung transplantation (population). We also aim to identify any harms that occur as a result of an exercise intervention.

Despite the link between frailty and poor outcomes after LTx,^17^ to our knowledge, this is the first systematic review of this topic. This review is important to evaluate the existing evidence, consider recommendations for clinical practice, identify gaps in the evidence base, and propose future research.

## METHODS

The review protocol was registered with PROSPERO (https://www.crd.york.ac.uk/prospero/display_record.php?RecordID=363730) and published prospectively.^18^ Reporting is according to Preferred Reporting Items for Systematic Review and Meta-Analysis (PRISMA) guidelines.^19^ The authors agree with and confirm that this study adheres to the principles of the World Medical Association Statement on Organ and Tissue Donation, the Declaration of Helsinki, and the Declaration of Istanbul. Ethical approval was not required due to this being a systematic review using previously published data.

After consulting with a medical information specialist, we searched MEDLINE (Ovid) 1980 to date, Embase (Ovid) 1980 to date, CINHAL Plus (EBSCO) 1980 to date, Cochrane Central Register of Controlled Trials (CENTRAL), and the Cochrane Library and trials registries (ClinicalTrials.gov and the WHO trials portal). Databases were searched from 1980 and last updated on February 21, 2024 (see Appendix 1 for search strategies, http://links.lww.com/CPTJ/A30). The success of LTx was established only after the introduction of the immunosuppressive agent cyclosporine, which became accepted practice in the early 1980s.^20^

We accepted studies pertaining to adults listed for single or double LTx with any underlying lung disease. Acceptable interventions included any formal physical exercise or activity prescribed under professional guidance, in any setting, with no minimum length or intensity. We included single exercise interventions, multicomponent or multi-modal programs. Types of studies included were any comparator or no comparator, but we anticipated no intervention, usual care, or advice only. Primary outcomes of interest were validated frailty or surrogate physical frailty measures. Where studies reported a relevant primary outcome, we considered the following as secondary outcomes: mortality (on waiting list or post-operatively), hospital or intensive care length of stay, and health-related quality of life measures. We recorded any adverse event reporting. Owing to a paucity of randomized controlled trials during preliminary searching, we included any primary research study design, including those without controls, with more than 10 participants. Full inclusion and exclusion criteria are detailed in Appendix 2, http://links.lww.com/CPTJ/A31.

Following deduplication in Endnote, title, abstract, and full-text screening were performed by L.M. and G.N. or another reviewer independently using Rayyan software.^21^ Any discrepancies were resolved by consensus. Non-English language studies were retained and listed for reference^22,23^ but not included in the synthesis process. Reference lists of review articles were checked for further relevant studies and abstracts checked to identify any later published in full text.

A standardized, piloted data extraction form was used to collect data relating to study design, participant characteristics, intervention details based on the Template for Intervention Description and Replication Checklist (TIDieR)^24^ and the Consensus on Exercise Reporting Template (CERT)^25^ and primary and secondary outcomes. We extracted characteristics that stratify health opportunities and outcomes (PROGRESS-plus)^26^ and other relevant data including funding sources, conflicts of interest, recruitment failure, and any patient and public involvement or engagement.^18^ For continuous outcomes, we extracted means with standard deviations (s.d.) for each group or medians with interquartile ranges (IQR) where reported. Mean differences or standardized mean differences with 95% CI were extracted where these were the only reported data. *P* values were extracted in the absence of other outcome data as was any descriptive reporting of results. Data extraction was completed by one author (L.M.) and checked by a second (G.N. or L.G.). Where possible, we extracted and reported any definitive statements regarding ethical procurement of donor organs. Where a study reported 2 cohorts of participants completing different interventions, without comparison, we reported each cohort with results separately (full data extraction detailed in Appendix 3, http://links.lww.com/CPTJ/A32).

Assessment of the study quality was performed by two researchers independently (L.M. and G.N. or L.G.) with discrepancies agreed by consensus, using the National Institutes of Health (NIH) tool for before–after (pre–post) studies without control groups.^27^ Meta-analysis was not possible due to heterogeneity of study interventions and outcomes with inconsistent reporting of effect measures and data across studies. A narrative synthesis was performed following the Synthesis Without Meta-analysis (SWiM) guidance.^28^ We performed vote counting using the direction of effect, without consideration of statistical significance, size of effect, or the minimally clinically important difference.^29^

## RESULTS

**Fig. 1.**
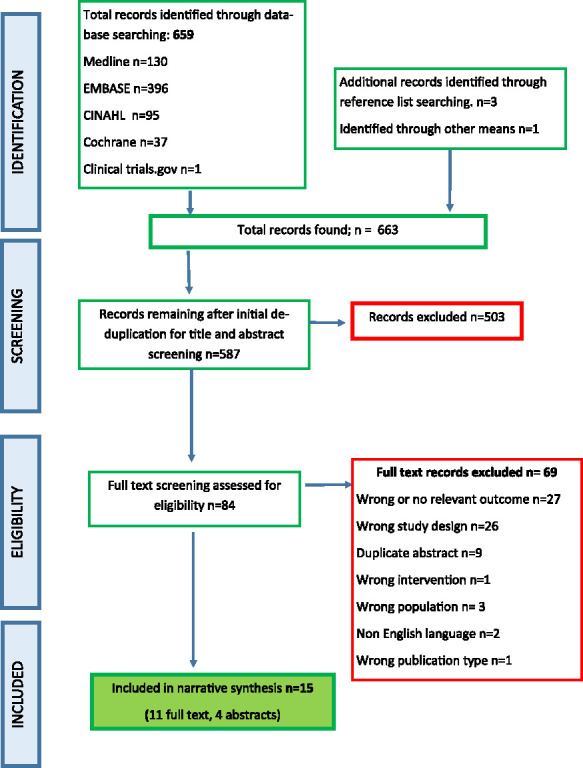
PRISMA flow diagram for searching and study selection process.

A total of 659 articles were identified from the database searches, 3 from handsearching reference lists and one through communication with colleagues. After deduplication and title and abstract screening, 84 records underwent full-text screening and assessment for eligibility. Of these, 22 records of 15 studies met the inclusion criteria for the review. (Fig. 1). They included 13 pre–post designs^30-42^ and one noninferiority study (see Table 1 for study characteristics).^43^ Where studies were otherwise relevant but reported no frailty outcomes, we made every attempt to contact authors to ascertain if these outcomes were measured but not reported. One randomized controlled trial (RCT) was included on this basis for completeness.^44^ Wickerson et al (2023)^40^ reported 2 cohorts, undertaking different interventions. No comparison was made between the exercise outcomes; therefore, the cohorts were reported as 2 separate groups for the purpose of this review.

**TABLE 1:** Characteristics of Included Studies

Author, Date, Reference	Study Design	Country	Population Size/Age/Gender/Diagnosis	Setting	Intervention	Duration and Frequency	Comparator	Primary Outcomes	Secondary Outcomes
Kambur et al (2017)^30^	Pre–post	Turkey	n = 21 Median age 36 years (IQR 15–68) 52% male	Hospital and home based	Pulmonary rehabilitation	Twice a week at hospital, 3 home sessions for 8 weeks	n/a	10 m walking time	None reported
Wickerson et al^31^ (2021)	Cohort observational Pre–post	Canada	n = 78 recruited^a^ n = at least 26 for each outcome Mean age 59 years (12) 47% male 50% ILD, 35% COPD, 1% CF, PH 7%, 2% bronchiectasis, 5% retransplant	Home based	App-based remote rehabilitation including aerobic and resistance training	At least 3 times a week, minimum of 4 weeks	n/a	SPPB (n = 42) Treadmill speed (n = 26) Quadriceps weight (lb) (n = 37)	None reported
Kerti et al^32^ (2021)	Cohort observational Pre–post	Hungary	n = 63 Mean age 58 years (6.6) 53% male 63% COPD, 29% IPF, 6% bronchiectasis, 2% alveolitis fibrosing	In-person group training	Breathing, strength and endurance exercises (high-intensity continuous or interval training)	30 mins daily breathing work, endurance work 15–20 mins, 2–3 times a day for 4 weeks	n/a	Handgrip strength	None reported
Pehlivan et al^44^ (2018)	RCT	Turkey	Intervention n = 17 Mean age 39 years (12) 64.7% male alveolar proteinosis 5.9%, CF 5.9%, ILD 11.8%, silicosis 11.8%, sarcoidosis 5.9%, RA lung disease 5.9%, bronchiectasis 35.3%, COPD 17.6%	In-person group PR IMT: Unsupervised home based	Pulmonary rehabilitation plus IMT	PR: 2 days a week for 3 months IMT	PR only n = 17 Mean age 36 (15.86) 58% male	Measured but not reported (confirmed by author contact)	None reported
Schneeberger et al^33^ (2020)	Prospective, observational cohort study Pre–post	Germany	n = 32 recruited n= 28 analyzed with complete data Mean age 60 years (5) %Male not reported COPD and ILD (no % reported)	In-patient PR program	PR	Not reported	n/a	SPPB	None reported
Kennedy et al^34^ (2018)	Prospective, observational cohort study Pre–post	United States	n = 63 Median age 65 years 60% male Diagnoses not reported	Not specified	PR	Not reported	n/a	Frailty phenotype Gait speed over 15 feet Handgrip strength	None reported
Al Ghofaily^35^ (2022)	A single center prospective cohort interventional study Pre–post	Saudi Arabia	n = 20 Mean age 58 years (9) % Male not reported	Not specified	PR Structured exercises according to guidelines from AACVPR	8 weeks	n/a	TUG	None reported
Pehlivan et al (2020)^36^	Pre–post	Turkey	n = 47 Mean age 39.38 years (14.56) 66% male Diagnosis: alveolar proteinosis (2.1%), bronchiectasis (38.3%), ILD (10.6%), Kartagener syndrome (2.1%), CF (10.6%), COPD (23.1%), RA lung involvement (2.1%), sarcoidosis (4.3%), silicosis (6.4%)	In-person group PR	Aerobic and strength program	3-month program Frequency not reported	n/a	Handgrip Quadriceps force	Not reported
Pehlivan et al^37^ (2018)	Pre–post	Turkey	n = 39 Mean age 36.89 years (13.41) 64% male Diagnosis: bronchiectasis (41%), emphysema (5%), silicosis (15%), ILD (12%), sarcoidosis (5%), COPD (10%), CF (10%)	In-person group PR and home exercise	PR: Aerobic and strength program with additional education component (dyspnoea management, bronchial hygiene, medications) Home exercise: breathing exercises, strengthening, and walking	2 days in-person PR, 3 days unsupervised home exercise for 8 weeks	n/a	Quadriceps force Biceps strength	SF-36
Wickerson et al^38^ (2020)	Retrospective pre–post	Canada	n = 150 listed for transplant n = 62 (with analyzed complete data) Median age 62 years (IQR 56–67) 53% male of those listed Of the 62 with all data, 60% ILD.	In-person PR	PR: strength and aerobic	90 minutes, 3 times a week from listing until transplant. Outcomes measured at 6 weeks	n/a	SPPB 4 m gait speed 5STS Balance component of SPPB	Not reported
Singer et al^39^ (2018)	Pilot, feasibility pre–post design	United States	n = 15 enrolled n = 13 analyzed with complete data Mean age 62.9 years (5.7) of enrolled 67% male of enrolled Fibrosis 10 (67%); COPD 5 (33%) Only enrolled those with SPPB ≤11	Home, app-based intervention (after in-person assessment and training phase). Weekly phone calls	(education in training phase; oxygen titration and dyspnoea management). Aerobic/strength exercise and nutrition intervention through app	Daily walking, 3 times a week app-based exercises for 8 weeks	n/a	SPPB FFP (modified) Handgrip strength	Not reported
Wickerson et al^40^ (2023)	Pre–post	Canada	Telerehab n = 23 age: median 61 years (IQR 54–69) 57% male ILD (50%), COPD (46%), CF (4%) In-person n = 26 Median age 61 years (IQR 56–61) 65% male ILD (74%), COPD (22%), PH (4%)	Telerehab through app Or In-person exercise	Telerehab through app and in-person: aerobic, resistance training, functional exercises, and flexibility Telerehab app-guided asynchronously by physiotherapist	Telerehab: Minimum 3 days a week In-person: 90 minutes, twice a week from listing until 3 months post-transplant	n/a	SPPB 4 m gait speed Quadriceps torque NB. Outcomes reported at 12 weeks post-transplant	ICU length of stay Acute hospital length of stay
Byrd et al^41^ (2022)	Pre–post study	United States	n = 57 enrolled n = 39 analyzed with complete data Mean age 50 years (16.1) 44% male Diagnosis: restrictive lung disease (38%), obstructive disease (23%), pulmonary vascular disease (5%), CF (26%), retransplant (8%)	In-person group and individual exercise	Aerobic (walking), strength, balance, breathing, and flexibility exercises	2.5 hours a day, 5 days a week, for 1 month	n/a	SPPB 4 m gait speed 5STS Leg press/leg extension/arm curls: change in resistance-lifted and volume-lifted Fullerton advanced Balance (FAB) Scale, the Short Form FAB (SF-FAB) Scale, and the Four Square Step Test (FSST) Instrumented balance assessment (postography) modified clinical test of sensory interaction with Balance (mCTSIB) and the limits of stability test	Not reported
Bourgeois et al^42^ (2024)	Pre–post study	Canada	n = 20 enrolled n = 14 analyzed after intervention n = 5 analyzed individually after maintenance period Mean age 57.9 years (11.0) 70% male Diagnosis: ILD (45%), COPD (30%), CF (10%), retransplant (5%), PAH (5%), scleroderma (5%)	Home based	Intervention phase: 1:1 video supervised strengthening and independent aerobic exercise Maintenance phase: Independent aerobic and strengthening	Intervention phase: 12 weeks, strength 3/week x 30 mins. Aerobic: 5/week (independent). Phase out of supervision = 3 sessions wk 1–4, 2 sessions week 5-8, 1 session (weeks 9–12)	n/a	SPPB 5STS 4mgs SPPB balance score	QOL—St George's respiratory questionnaire
Byrd et al^43^ (2024)	Noninferiority study with pre–post data for both groups	United States	Individual exercise group: n = 81 56.8% male median age 65 years (IQR 58, 70) obstructive 21 (25.9%), vascular 2 (2.5%), cystic 5 (6.2%), restrictive 53 (65.4%) Group exercise: n = 93 54.8% male median age 62 years (IQR 48–68) Obstructive 24 (25.8%), vascular 2 (2.2%), cystic 8 (8.6%), restrictive 59 (63.4%)	In-person at transplant centre	Individual exercise group 1:1 face-to-face exercise aerobic, strengthening, video-conferencing education	4–5 weeks, 5 days a week Individual: daily 40 mins aerobic, upper/lower limb strengthening, 1 session on/off floor, virtual education	Group exercise: aerobic, strengthening, balance, flexibility, education, diaphragmatic breathing Group: 4–5 weeks, 5 days a week, daily 40–50 mins aerobic, upper or lower limb strengthening, flexibility, balance, 30 mins class plus education	SPPB	Hospital LOS QOL—the Ferrans and Powers quality of life index Pulmonary version III (QLI)

a Number of participants with full analyzed data varies by outcome.

AACVPR, American Association of Cardiovascular and Pulmonary Rehabilitation; TUG, Timed up and Go test; COPD, chronic obstructive lung disease; ILD, interstitial lung disease; IPF, idiopathic pulmonary disease; CF, cystic fibrosis; RA, rheumatoid arthritis; PH, pulmonary hypertension; SPPB, Short Physical Performance Battery; ICU, intensive care unit; PR, pulmonary rehabilitation; SF-36, 36 Item short form survey; 5STS, 5 times sit-to-stand test; QOL, quality of life; LOS, length of stay.

### Quality Assessment

Studies demonstrated low-to-fair methodological quality (Table 2). In the 4 studies which were only reported in abstract form,^30,33-35^ the assessment of study quality was hindered due to lack of information. Lack of reporting regarding intervention content and replicability was a common feature. Lack of blinding of outcome assessors and insufficient sample size were the main factors affecting the study quality (Table 2). Studies reported data without the use of intention to treat analysis, instead presenting a completed case analysis. Where subject attrition reasons were reported, primary reasons included transplantation, delisting, death, or drop-out with no further explanation.

**TABLE 2:** Risk of Bias of Included Studies

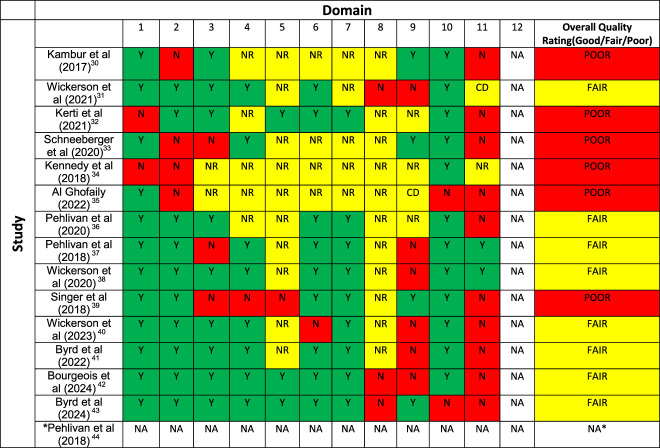

Yes (Y), No (N), Cannot determine (CD), Not applicable (NA), Not reported (NR). Overall rating: Good, Fair, or Poor.

*Article met inclusion criteria; author contact highlighted frailty outcomes measured but not reported in published article.

Domains:

1. Was the study question or objective clearly stated?

2. Were eligibility/selection criteria for the study population prespecified and clearly described?

3. Were the participants in the study representative of those who would be eligible for the test/service/intervention in the general or clinical population of interest?

4. Were all eligible participants that met the prespecified entry criteria enrolled?

5. Was the sample size sufficiently large to provide confidence in the findings?

6. Was the test/service/intervention clearly described and delivered consistently across the study population?

7. Were the outcome measures prespecified, clearly defined, valid, reliable, and assessed consistently across all study participants?

8. Were the people assessing the outcomes blinded to the participants' exposures/interventions?

9. Was the loss to follow-up after baseline 20% or less? Were those lost to follow-up accounted for in the analysis?

10. Did the statistical methods examine changes in outcome measures from before to after the intervention? Were statistical tests done that provided *P* values for the pre-to-post changes.

11. Were outcome measures of interest taken multiple times before the intervention and multiple times after the intervention (i.e., did they use an interrupted time-series design)?

12. If the intervention was conducted at a group level (e.g., a whole hospital, a community, etc.) did the statistical analysis take into account the use of individual-level data to determine effects at the group level?

### Certainty of Evidence

A GRADE assessment was completed by outcome (Table 3). Evidence was very low to low certainty for all outcomes and was downgraded due to risk of bias, inconsistency due to participant heterogeneity, and imprecision due to low number of participants in few studies with wide confidence intervals. Summaries of effects by outcome are provided in Table 4, and the direction of effects by outcome per study are demonstrated in Table 5.

**TABLE 3:** Outcome Mapping by Intervention Type Including GRADE Certainty of Evidence Classification

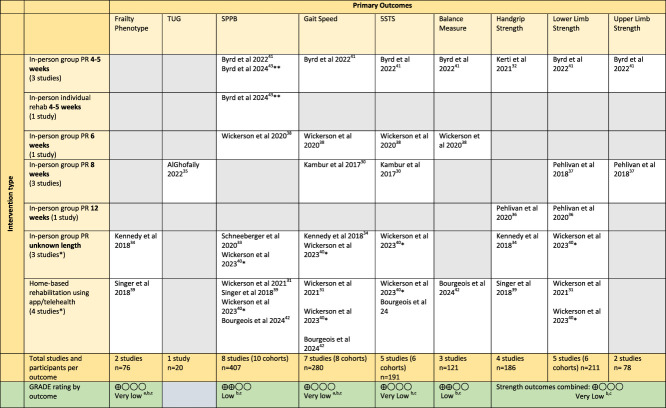

**TABLE 4:** Effects of Exercise Training on Outcome Measures

Author, Y	Study Design	n	Time Point (wk)	Measure	Mean Difference/Standardized Mean Difference (95% CI)	Pre–post *P*	Effect Size
Wickerson et al, 2021^31^	Pre–post	78	4	SPPB	NR	0.9	NR
Schneeberger et al, 2020^33^	Pre–post	32	Unknown	SPPB	Mean difference +1.4 (0.95–1.8)	<0.001	NR
Al Ghofaily^35^, 2022	Prospective pre–post	20	8	TUG	Mean difference 1.79 (0.45) seconds	NR	NR
Wickerson et al^38^, 2020	Retrospective pre–post	62	6	SPPB	NR	Whole group 0.01 frail/prefrail group <.001 not frail group 0.9	NR
Singer et al, 2018^39^	Pilot, feasibility pre–post design	13	8	SPPB	Mean change 1.0 (1.9)	0.08	NR
Wickerson et al, 2023^40^	Pre–post	23 + 26	12 postoperative	SPPB	NR	In-person *P* = .18 telerehab *P* = .25 whole group *P* = .08	NR
Byrd et al^41^ 2022	Pre–post	39	4	SPPB	Mean difference 0.38 (SEM 0.13) (0.12–0.65)	0.05	0.54
Bourgeois et al^42^ 2024	Pre–post	20	12	SPPB	Mean change 0.4 (−0.1–0.9)	0.059	0.56
Byrd et al^43^ 2024	Noninferiority study. Pre–post data	81 + 93	4–5	SPPB	Individual exercise mean change 0.4 (0.2, 0.7) group exercise mean change 0.4 (0.1, 0.7)	NR	NR
**Effects of exercise training on gait speed measures**							
Kambur et al, 2017^30^	Pre–post	21	8	10 m walk time (s)	NR	≤0.001	NR
Wickerson et al, 2021^31^	Cohort observational Pre–post	78	4	Treadmill speed	NR	0.31	NR
Kennedy et al, 2018^34^	Prospective, observational cohort study Pre-post	63	Unknown	15 feet walk time (s)	NR	0.008	NR
Wickerson et al, 2020^38^	Retrospective pre–post	62	6	4 m gait speed (m/s)	NR	0.25	NR
Wickerson et al, 2023^40^	Pre–post	23 + 26	12 postoperative	4 m gait speed (m/s)	Telerehab: Median change 0.21 [0.11, 0.47) in-person: 0.04 [−0.08–0.35] whole group change 0.16 (0.06, 0.32)	Telerehab <0.001 in-person 0.13 whole group 0.0001	NR
Byrd et al, 2022^41^	Pre–post study	39	4	4 m gait speed (m/s)	Mean diff 0.01 (SEM 0.03) 95% CI -0.05 - 0.07	0.735	0.19
Bourgeois et al^42^, 2024	Pre–post study	20	12	4 m gait speed (scored as part of SPPB 0–4)	Mean difference 0.1 (−0.1 - 0.2)	0.317	0.22
**Effects of exercise training on timed sit to stand measures**							
Kambur et al 2017^30^	Pre–post	21	8	5STS	NR	<0.001	NR
Wickerson et al^38^ 2020	Retrospective pre–post	62	6	5STS	NR	0.007	NR
Wickerson et al, 2023^40^	Pre–post	23 + 26	12 post operative	5STS	Telerehab: Median change 0.26 [−1.23–3.31] in-person median change 0.84 [−0.15–2.2] whole group change 0.50 (−0.17, 2.04)	Telerehab *P* = .39 in-person *P* = .08 whole group *P* = .07	NR
Byrd et al^41^, 2022	Pre–post	39	4	5STS	Mean diff −1.31 (SEM 0.34) (−1.99 - 1.62)	<0.001	0.48
Bourgeois et al^42^, 2024	Pre–post	20	12	5STS	Mean change −1.4 (−2.3 to −0.5)	0.009	0.61
**Effects of exercise on strength outcomes**							
Wickerson et al, 2021^31^	Cohort observational Pre–post	78	4	Quadriceps weight (lbs)	NR	0.08	NR
Kerti et al, 2021^32^	Cohort observational Pre–post	63	4	Handgrip strength	NR	“Not significant”	NR
Kennedy et al, 2018^34^	Prospective, observational cohort study Pre–post	63	Unknown	Handgrip strength	NR	“Not significant”	NR
Pehlivan et al, 2020^36^	Pre–post	47	12	Handgrip strength Quads force	NR NR	<0.0001 0.094	NR NR
Pehlivan et al, 2018^37^	Pre–post	39	8	Quadriceps force (lb) Biceps strength (lbs)	NR NR	0.95 0.32	NR NR
Singer et al, 2018^39^	Pilot, feasibility pre–post design	13	8	Handgrip strength	NR	0.48	NR
Wickerson et al, 2023^40^	Pre–post	23 + 26	12 post operative	Isometric quadriceps strength	Telerehab median change −9.6 [−2.9 to −2.3] in-person median change: −1.6 [−2.5–8.1] whole group change −1.23 (−12–3.7)	Telerehab *P* = .02 in-person *P* = .79 whole group *P* = .13	NR
Byrd et al, 2022^41^	Pre–post study	39	4	leg press weight (lb) and volume (weight x repetitions) leg extension weight (lb) and volume (weight x repetitions) bicep curl weight (lb) and volume (weight x repetitions)	Weight MD: 15.07 (SEM 1.69), 95% CI 11.61 - 18.51 volume MD 668.83 (SEM 77.22), 95% CI = 510.89–826.77 change in weight leg extension: 8.71(SEM1.44), 95% CI = 5.79–11.63 change in volume leg extension: 336.32 (SEM 46.12), CI 242.86 - 429.77 change in weight arm curl: 3.46 (SEM 0.71), CI 2.01, 4.91 change in volume arm curl: 74.04 (SEM 10.89), CI 51.98, 96.10	Weight *P* < .001 volume *P* < .001 Weight *P* < .001 volume *P* < .001 Weight *P* < .001 volume *P* < .001	Weight 1.13 volume 1.92 Weight 0.71 volume 1.29 Weight 1.29 volume 1.23

Author, y	Study Design	n	Time Points (wk)	Measure	Mean Difference/Standardised Mean Difference (95% CI)	Pre-post *P*	Effect Size	Other
**Effects of exercise training on frailty phenotype measures**								
Kennedy et al, 2018^34^	Prospective, observational cohort study Pre–post	63	Unknown	FFP	NR	NR	NR	43.5% of patients' frail at baseline improved their FFP score following PR
Singer et al, 2018^39^	Pilot, feasibility pre–post design	13	8	FFP (modified)	Change −0.6 (1.0)	.07	NR	NR

5STS, 5 times sit-to-stand test; FFP, fried frailty phenotype; NR, not reported; SPPB, Short Physical Performance Battery; TUG, timed up and go.

**TABLE 5:** Direction of Effects

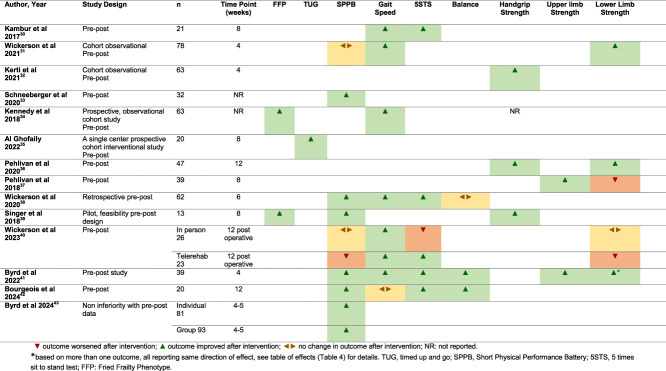

### Interventions

There was considerable heterogeneity in interventions which mostly comprised in-person group PR including aerobic and strengthening components. Digital interventions appeared in more recent studies, including video-guided strengthening and app-based interventions (see Table 1 for characteristics of studies). Additional interventions incorporated into rehabilitation programs included flexibility, breathing exercises, and balance training.

Only one study reported the inclusion of specific frailty-targeting intervention exercises based on the Strong For Life and Weight Bearing Exercise for Better Balance programs.^39^ This app-based study was the only multicomponent study with intervention and support with nutrition from a registered dietitian.

Duration of interventions varied from 4 weeks^32,41,43^ to 12 weeks.^36,42^ Wickerson et al^40^ (2023) was the only study to continue the intervention, and measure outcomes 12 weeks after transplantation had occurred. The frequency of interventions varied from twice a week in-person sessions plus 3 home sessions a week,^30^ to daily supervised exercise sessions.^32^ Most studies expected an independent exercise component alongside the planned intervention but reporting of adherence to this was rare. Direct comparison of intervention components was not possible due to lack of reporting detail in some studies (see Table 1). Table 3 maps all studies and demonstrates the heterogeneity of both outcomes reported and interventions completed.

### Frailty Outcomes

Only 2 studies used frailty phenotype as a primary outcome (n = 76, very low certainty evidence).^34,39^ Kennedy et al^34^ (2018) reported an improvement in 43.5% of the patients deemed frail by FFP at baseline following PR, although the specific intervention, frequency, and duration are unclear due to limited reporting in abstract form. Singer et al. (2018) used a modified FFP which had previously been shown to have a better predictive and construct validity in LTx candidates than the original FFP.^45^ In their small, 8-week pilot study, they found that daily walking and 3 times a week app-based exercise and nutrition intervention showed an improvement in frailty (*P* = .07).^39^ One small, in-person study of PR demonstrated improvements in the Timed up and Go (TUG) measure.^35^

The most commonly reported outcome was the Short Physical Performance Battery (SPPB) (8 studies, n = 407, low certainty evidence). Seven cohorts showed a preoperative improvement (Table 5). Wickerson (2020) investigated SPPB score change by baseline SPPB status and found that the group of patients deemed frail or prefrail at baseline had a significant improvement after in-person PR (*P* = .001) compared with the group who were not frail at baseline (*P* = .9). They defined prefrail to be SPPB ≤ 9 for the purpose of this study.^38^

### Surrogate Frailty Outcomes

Four different measures of gait speed were reported ranging from speed over 4 to 10 meters plus treadmill speed (7 studies, n = 280, very low certainty evidence). Gait speed increased in all except one study (Table 5). None of the shorter 4 to 6 week studies showed a significant improvement; however, improvements were seen in an 8-week program (*P* < .001)^30^ and an undisclosed duration of PR which increased walk time over 15 feet (*P* = .008).^34^

Outside of the balance component of the SPPB, only one study examined balance using a comprehensive range of functional balance scales plus an instrumented balance assessment.^41^ Significant improvements in Fullerton Advanced Balance (FAB) Scale (*P* < .001), the Short Form FAB (SF-FAB) Scale (*P* < .008), and the 4 Square Step Test (FSST) (*P* < .019) balance scores were observed after an intensive, multicomponent 4-week program of exercise although certainty of evidence was low (Table 3). Timed sit-to-stand outcomes showed significant postintervention improvements in 4 of 6 cohorts^30,38,41,42^ which included both in-person and remote digital interventions; however, certainty of evidence was very low (Tables 2 and 3).

### Strength Outcomes

Handgrip (4 studies, n = 186) was the most commonly measured upper limb strength outcome with 3 studies reporting an improvement (very low quality of evidence). This is a commonly used surrogate measure of overall body composition and frailty in LTx.^46^ Pehlivan et al (2020) reported the only statistically significant improvement in handgrip strength (*P* < .0001) after a 12 week in-person PR program, which included arm ergometry.^36^

There was considerable heterogeneity in lower limb strength measurement. Quadriceps measures were the most commonly reported (5 studies, n = 211); however, their relationship to functional and frailty-specific measures is unclear. There was evidence of lower limb strength improvements in 3 studies.^31,40,41^ Wickerson et al^31^ (2021) found increases in quadriceps weight achieved yet failed to demonstrate changes in SPPB scores. Two cohorts experienced a nonsignificant reduction in quadriceps strength,^37,40^ and one did not change with the intervention^40^ (Table 5), although 2 of these cohorts had outcomes measured 12 weeks post-LTx,^40^ and overall certainty of evidence was very low (Table 3). Despite showing improvements in functional or frailty measures, 8 studies failed to measure the lower limb strength.^30,33-35,38,39,42,43^

Byrd et al.^41^ (2022) found their 4-week exercise program to have significant improvements in both leg press and leg extension outcomes (both *P* < .001). They found improvements in 5 times sit-to-stand test (5STS) scores, which have been linked to increase in leg strength.^40^ They also demonstrated improvements in gait speed, SPPB scores, and balance metrics, all of which are recognized functional and surrogate frailty indicators. Although the link here between leg strength and frailty measures and surrogates appears promising, direct causation cannot be assumed and is yet to be clearly demonstrated in this population.

### Secondary Outcomes

Secondary outcomes were only reported in 3 studies, and no significant improvements were detected, despite a range of intervention types and durations of 4 to 12 weeks.^37,42,43^ Two studies measured postoperative length of stay (LOS), of which no significant difference between intervention types was noted.^40,43^

### Adherence

Adherence, defined here as percentage of prescribed sessions completed, was only reported in 3 app/telehealth studies. When measured through digital records of sessions completed, adherence ranged from a mean of 60%^39^ to 91.9%^42^ although poor completion of paper diaries prevented independent exercise being assessed.

### Adverse Events

Where adverse events were reported, (in 3 remote digital and one face-to-face intervention), no complications or events occurred.^31,37,39,42^ A summary of study effects by outcome is displayed in Table 4.

## DISCUSSION

This review has systematically searched and synthesized the evidence for the effect of exercise interventions on physical frailty in 15 studies of 664 individuals awaiting LTx. This review has demonstrated that despite some evidence of positive effects of in-person and remote digital aerobic and strength training on physical frailty outcomes, current evidence is limited to uncontrolled pre–post designs and has low to very low certainty across outcomes.

### Heterogeneity in Studies

The low to very low certainty of evidence from studies in this review is influenced by the lack of adequately powered RCTs and heterogeneity in this population (Table 1). This may be due to the rarity of LTx, single center studies, and the complexity and unpredictability of the waiting list period. The ethical implications of studies with a nonexercising control group are a likely barrier to RCTs. Pulmonary rehabilitation has robust evidence for improving exercise capacity and quality of life in this population^12^ and is well-recognized as part of standard care.

There was heterogeneity of studies in terms of population age, which varied from a median 36 (IQR 15–68)^30^ to a median age 65 years (IQR 58, 70),^43^ and where reported, underlying disease type, which affects the ability to compare results between studies. Frailty is not directly associated with age in the LTx candidate group,^47^ but the disease mechanism, course, medication, and other bodily systemic effects vary widely from idiopathic pulmonary fibrosis to cystic fibrosis (CF), for example. Evidence of validity across the range of frailty outcomes in different lung disease groups is also unclear.^48^ LTx candidates with CF have demonstrated high levels of frailty when measured by Frailty Index measures^49^ yet have been observed to be significantly less frail than those with mixed disease and COPD with the use of the SPPB.^50^ Despite the well-documented differences in pathophysiology and presentations of the different lung diseases, there are no disease-specific guidelines on addressing frailty through exercise training. Balance and gait disturbances are recognized components of frailty and are common in people with COPD.^48^ It may be that addressing disease-specific frailty components helps to personalize rehabilitation for different disease groups, although further guidance is needed in the literature.

Lengths of interventions varied from 4 to 12 weeks (Table 1), which implies a difference in total exercise doses.^51^ While more exercise may produce stronger benefits, paradoxically longer interventions provide more time for an increase in disease severity progression and repeated exacerbations, both of which may affect the ability to exercise and demonstrate improved functional outcomes. A previous systematic review on exercise in solid organ transplant candidates reported improvements in programs over 10 weeks in duration,^52^ but longer programs can result in participant attrition due to waiting list mortality or participants undergoing LTx. Effect sizes were similar for changes in SPPB after 4 and 12 weeks interventions^41,42^ although the different program content and mode of delivery prevents direct comparison. Studies showed a significant increase in lower limb strength in as little as 4 weeks (Table 4).^31,41^

### Reporting of Outcome Measures

There is a notable variation in frailty and surrogate measures across the studies (Tables 1 and 3). This is potentially due to the lack of consensus of a core outcome set for exercise studies of LTx candidates^53^ and absence of recommendations for physical frailty measurement during assessment for LTx.^1,53^

There is limited use of surrogate physical frailty measures such as hand grip (n = 4) and balance (n = 3). Handgrip has been shown to be strongly associated with quadriceps strength^47^ and functional performance^46^ in LTx candidates and is a commonly used, quick, reliable, simple, inexpensive test reflective of frailty performed with other preoperative populations.^54^ Handgrip measurement is recommended in the evaluation of pre-LTx and post-LTx rehabilitative needs^46,55^ yet remains sparsely utilized in these intervention studies (Table 3). Reduced levels of lower limb muscle strength are associated with frailty in older adults,^56,57^ yet the evidence for association in the LTx population is sparse, in part due to the lack of controlled studies reporting both lower limb and frailty outcomes.

Gait speed measures varied between studies (Table 1) but were generally measured over distances under 10 m. The potential for variation in patient instruction and the impact of acceleration and deceleration phases over short distances are unknown. There may therefore be implications for variation in results as a function of the method of testing rather than true effects of an intervention.

The deleterious effects of frailty on postoperative outcomes have been identified in other surgical populations.^58^ There was a dearth of outcomes related to postoperative recovery; therefore, the impact of preoperative rehab and improvements in frailty on the recovery from LTx remain unclear. This could be due to the complexity of confounders related to perioperative recovery and the variability of time on the waiting list increasing the logistical complexity of data collection.

### Intervention Components

Despite frailty being measured in the studies within this review, to what extent the interventions were developed with physical frailty in mind is unclear. Interventions mostly comprised aerobic and strength training. In older adults, strength, flexibility, and balance components are highlighted as important interventions to modify or prevent frailty.^59^ Multimodal interventions, such as those targeting exercise, nutrition, and psychological support, are better able to address the interplay between physical and psychological factors, and this approach is known to positively influence the outcomes of interventions.^60^ Pilot data from the only combined nutritional and exercise intervention in this review look promising,^39^ but further well-powered studies are required.^61^

The exercise interventions prescribed in this review may be clinically appropriate for this breathless population, but elements of progression were impacted by patient autonomy (particularly home-based programs) alongside tailoring for each individual and were therefore complex and unstandardized. While personalized training is considered essential,^51^ this form of intervention is hard to report and replicate and is affected by many dependent factors such as experience of clinicians^62^ and protocols in each institution alongside safety considerations, such as policies for exercise desaturation and oxygen prescription. This is a phenomenon previously reported in studies of exercise in the LTx pathway.^63^ While pragmatic in design, replication is challenging. Researchers and clinicians require detailed descriptions of the applied procedures. Reliable documentation using a variety of subjective and objective instruments and tools such as TIDieR^24^ would increase the quality of reporting,^51^ particularly with poor adherence being a key issue with the management of chronic health conditions.^63^ Limited reporting of both intervention components and adverse events within the included studies limits the conclusiveness of safety of these programs. Similarly, transparency of the definition of adverse events in each study is essential for clinicians considering the application of interventions to their waiting list population.

### Intervention Context: Remote Versus In-Person

While pandemic-imposed restrictions may have affected some study outcomes, they also provided an opportunity with an imposed shift toward telerehabilitation and app-based remote interventions. Frailty is an independent predictor of noncompletion for in-person PR in those with COPD.^14^ App-based PR has been shown to improve exercise capacity and quality of life when compared with conventional PR.^64^ Pilot studies of LTx candidates with a variety of underlying disease types have demonstrated good adherence and acceptability levels of remote, digital rehabilitation platforms.^39,42,65^ Digital alternatives for LTx prehabilitation appear appealing due in part to the significant travel distances required to access the nearest LTx center.^65^ Services such as virtual visits and remote digital monitoring could mitigate resource issues which can lead to worse LTx outcomes in low socioeconomic groups.^66^ Poor digital literacy and lack of access to appropriate, reliable devices and internet connectivity could negate those potential benefits however.^67^ Other reported barriers to home-based digital interventions include access to home exercise and monitoring equipment^31^ and poor adherence to remote monitoring devices such as activity trackers.^39^ Despite these reported barriers, Singer et al^39^ (2018) reported their customized mobile health technology, delivering exercise, and nutrition interventions and was capable of improving frailty in adult LTx candidates.

There is currently insufficient evidence to suggest a digital, in-person, or hybrid approach confers an increased benefit in frailty outcomes for this population. Adequately powered RCTs and the identification of the barriers and facilitators of different prehabilitation approaches are therefore required.^31,39,68^

### Implementation

Recognition of LTx prehabilitation as a complex intervention highlights the importance of the interaction between the intervention and its context. Variation in setting and provision of supervision, as well as specific exercise frequency, intensity, and type, will shape how outcomes are affected^62^ and remains a challenge. There is therefore a need for collaboration, involving patients as partners to support the design, delivery, and successful implementation of future studies while paying attention to the resources required, as well as impact on real-world implementation.^62,69^ The lack of reporting of PROGRESS-PLUS criteria (see Appendix 4, http://links.lww.com/CPTJ/A33) in the included studies means that socially stratifying factors are not transparent.^26^ Patients from minority and low socioeconomic groups are known to have reduced access to effective treatments of chronic lung disease such as PR.^70^ In people with chronic health conditions and COPD, increasing frailty is significantly associated with low socioeconomic status (education and income) and social support.^71,72^ The centralized transplant center system can lead to unrecognized inequities related to travel, and caregiver lost wage costs.^66^ These factors play a role in contributing to inequities in health outcomes and should be considered when evaluating research outcomes.^26^ The effects in this review are therefore unknown. This review was therefore unable to determine the impact that socially stratifying factors may have contributed toward rehabilitation uptake, adherence, and their effect on frailty outcomes.

Limitations of our review include a lack of RCTs. We excluded but retained 2, non-English language articles, and therefore, feel the impact on the review is minimal.^22,23^ It is possible that we may have missed some studies of individuals with chronic lung disease with relevant data, where the inclusion of LTx candidates was not specified. Owing to the strict eligibility criteria and therefore rarity of LTx within chronic lung disease populations, it is unlikely that this has substantively affected our review outcomes and conclusions. Data synthesis was limited due to heterogeneity and the uncontrolled and underpowered nature of the included studies with a variety of reported data. The use of vote counting based on the direction of effect, while felt to be an appropriate strategy, provides no information on the magnitude of effects and is less powerful than methods to combine *P* values.^29^

Strengths of this study include its rigorous methodology following a predefined protocol. Support from an information specialist ensured thorough and comprehensive search strategies. All review stages utilized 2 reviewers. Screening and inclusion of studies was performed by 2 investigators independently to help minimize bias.

## CONCLUSIONS

Exercise training, both in-person and remote, appears beneficial in modifying some markers of physical frailty before LTx. The certainty of evidence for effects of exercise training on physical frailty is low or very low for all outcomes due to imprecision and high risk of bias. High-quality, adequately powered RCTs are needed to determine the impact of exercise interventions and multimodal interventions on physical frailty before LTx alongside postoperative outcomes, and to develop guidelines for exercise prescription in this population. Future studies would benefit from a focus on the feasibility of blended interventions and factors affecting the adherence of prehabilitation before LTx.
